# The Mediating Effects of Alexithymia, Intolerance of Uncertainty, and Anxiety on the Relationship Between Sensory Processing Differences and Restricted and Repetitive Behaviours in Autistic Adults

**DOI:** 10.1007/s10803-021-05312-1

**Published:** 2021-10-13

**Authors:** Heather L. Moore, Samuel Brice, Lauren Powell, Barry Ingham, Mark Freeston, Jeremy R. Parr, Jacqui Rodgers

**Affiliations:** 1 School of Psychology, 4.28, Dame Margaret Barbour Building, Wallace Street, Newcastle Upon Tyne, NE2 4DR UK; 2 Population Health Sciences Institute, Sir James Spence Institute, Newcastle University, Royal Victoria Infirmary, Level 3, Queen Victoria Road, Newcastle Upon Tyne, NE1 4LP UK; 3 Cumbria, Northumberland, Tyne & Wear NHS Foundation Trust, St. Nicholas Hospital, Jubilee Road, Newcastle Upon Tyne, NE3 1XT UK

**Keywords:** Autism, Alexithymia, Intolerance of uncertainty, Anxiety, Sensory processing, Restricted and repetitive behaviours

## Abstract

Distress caused by sensory processing differences for autistic individuals may be reduced by repetitive behaviours (RRB), including repetitive motor (RMB) and insistence on sameness (ISB) behaviours. Intolerance of uncertainty (IU) and anxiety mediate the relationship between sensory processing and RRB in autistic children. We replicated this model in autistic adults, extending it to include alexithymia. Serial mediation, using data from 426 autistic adults, identified significant direct effects from sensory processing to RMB and ISB, and indirect effects through alexithymia-IU-anxiety for RMB, and IU alone, and alexithymia-IU for ISB. Different mechanisms may underpin RMB and ISB. Alexithymia alongside, IU and anxiety, should be considered when understanding the relationship between sensory processing and RRB, and when offering interventions to support autistic people.

**Supplementary Information:**

The online version contains supplementary material available at 10.1007/s10803-021-05312-1.

Core characteristics of autism include sensory processing differences and restricted and repetitive behaviours (RRB; American Psychiatric Association 2013). While research with autistic children suggests a direct pathway between sensory processing differences and RRB as well as an indirect path via intolerance of uncertainty (IU) and anxiety (Glod et al., 2019; Wigham et al., 2015), very little research has explored these pathways with autistic adults (Hwang et al., 2019). No research to date has explored the importance of alexithymia (difficulty identifying and describing emotions) within these relationships.

Autistic people may experience differences in the perception of sensory stimuli compared to non-autistic individuals, resulting in differing sensory thresholds and, at times, heightened arousal (Gal et al., 2002). Studies evidence high rates of extreme hyper or hypo-reactivity in autistic adults (Crane et al., 2009), as well as the persistence of sensory processing differences throughout the lifespan (Boyd et al., 2010; Gabriels et al., 2008; Grandin & Scariano, 1986; Kern et al., 2006, 2007; Kientz & Dunn, 1997; O'Brien et al., 2009; Williams, 1992). Measures of RRB are frequently reported to comprise two factors: Repetitive Motor Behaviours (RMB) and Insistence on Sameness Behaviours (ISB) (Cuccaro et al., 2003; Honey et al., 2012; Richler et al., 2007; Risi et al., 2006). RMB include repetitive language, movements, spinning round and around, or tapping on a surface over long periods, and may provide additional stimulation when the sensory threshold is underwhelmed (Baker et al., 2008; Kapp et al., 2019; Kientz & Dunn, 1997; Turner, 1999). ISB capture a preference for uniformity and consistency in the environment (e.g. invariance from routines and circumscribed interests) and may function to lower arousal by blocking further sensory input when the sensory threshold is exceeded (Zentall & Zentall, 1983). Research by Chen et al. (2009) and Glod et al. (2019) has indicated a significant relationship between the severity of sensory processing differences and the amount, frequency, and intensity of RRB in autistic children.

Co-occurring mental health conditions are commonly experienced by autistic people. A meta-analysis of studies including young autistic individuals showed that at least 40% experience anxiety (van Steensel et al., 2011), whilst a meta-analysis of studies including autistic adults found current and lifetime prevalence of anxiety to be 27% and 42%, respectively (Hollocks et al., 2019). Intolerance of uncertainty (IU), or the tendency to react negatively to unforeseen/unpredictable situations and events (Freeston et al., 1994), is a widely proposed transdiagnostic mechanism of anxiety in both typically developing and autistic populations (Buhr & Dugas, 2009; Jenkinson et al., 2020; Koerner & Dugas, 2008).

Importantly, sensory processing differences have been linked to both IU and anxiety in autistic populations. sensory processing differences are considered to be a significant source of anxiety for autistic people (South & Rodgers, 2017; White et al., 2009) and greater sensory processing differences are associated with higher levels of anxiety in samples of autistic toddlers, children, and adolescents (Ben-Sasson et al., 2008; Uljarević et al., 2016). Moreover, IU has been directly linked to sensory processing differences in autistic and non-autistic children after controlling for the effects of anxiety (Neil et al., 2016). Sensory processing differences may, therefore, lead to difficulties with uncertainty surrounding a situation, and consequently anxiety. In addition to sensory processing differences, engagement in RRB may also be associated with uncertainty-related anxiety, in the absence of sensory challenges. In this context, RRB, particularly ISB, may act as a mechanism to narrow the incoming sensory information in order to reduce uncertainty and consequently, anxiety (Joyce et al., 2017; Lidstone et al., 2014; Rodgers et al., 2012; Wigham et al., 2015).

Wigham et al. (2015) were the first to investigate the relationships between sensory processing differences, IU, anxiety and RRB, in a sample of 53 autistic children (mean age: 12.49 years). The authors reported significant direct relationships between sensory hypo-reactivity and both RMB and ISB; and between sensory hyper-reactivity and ISB, while controlling for age and gender. However, they reported no significant direct relationship between sensory hyper-reactivity and RMB. Serial mediation through IU to anxiety significantly explained the relationship between sensory processing differences and ISB behaviours, whereas the total indirect pathways between IU and anxiety explained both RMB and ISB. This study highlighted the potentially important roles played by IU and anxiety in sensory processing differences and RRB in autistic children. More recently, Glod et al. (2019) replicated this study in a sample of 19 autistic children, and found direct effects from both sensory hyper- and hypo-reactivity to RMB and ISB. Indirect effects were only found for the relationship between sensory hypo-reactivity and ISB through anxiety alone, and serially through IU and anxiety; however, the small sample may partially account for these results.

Partially replicating Wigham et al. (2015), Hwang et al. (2019) investigated the mediating effect of IU in the relationship between autism and anxiety, sensory processing differences and anxiety, and anxiety and RRB in a sample of 176 autistic adults. The authors did not investigate the direct relationship between sensory processing differences and RRB, or indirect effects involving anxiety, as explored by Wigham et al. (2015). IU was a significant mediator in all relationships, except the relationship between anxiety and RMB. This research emphasises the central role of IU in the relationships between anxiety and sensory processing differences and RRB in autistic adults.

The importance of IU and anxiety to the relationship between sensory processing differences and RRB may also be related to alexithymia. Alexithymia is characterised by a difficulty identifying and describing emotions (Nemiah et al., 1976), and is reported to be present in around 40–65% of autistic people (Allen et al., 2012; Bird & Cook, 2013; Hill & Berthoz, 2006; Kinnaird et al., 2020; Milosavljevic et al., 2016; Salminen et al., 1999). Research suggests that autistic people with alexithymia experience increased levels of sensory processing sensory processing difficulties (Milosavljevic et al., 2016) and anxiety (Bird & Cook, 2013; Milosavljevic et al., 2016; Oakley et al., 2020), and research has identified autism symptoms and alexithymia as significant predictors of IU (Maisel et al., 2016). Additionally, significant associations have been found between alexithymia and IU, and IU and anxiety, although not alexithymia and anxiety in autistic children and adults (Gaigg et al., 2020; Ozsivadjian et al., 2021). Alexithymia and emotional acceptance (the ability to accept internal experiences) (Maisel et al., 2016), alexithymia and emotion regulation (Morie et al., 2019), and alexithymia, IU, and sensory sensitivity (Pickard et al., 2020) have been identified as significant mediators of the relationship between autism symptoms and anxiety in autistic adolescents and adults. While IU was not a significant mediator, alongside alexithymia and emotional acceptance, Maisel and colleagues postulate that sensory processing differences and alexithymia may increase feelings of uncertainty, which may result in ISB. Reviewing the literature, Poquérusse et al. (2018) propose that sensory processing differences and alexithymia together may lead to difficulty understanding the sensory environment, through disruption in how physiological arousal modulates emotional experiences. In a non-autistic sample, Liss et al. (2008) identified alexithymia as a mediator of the relationship between sensory processing differences and anxiety. However, no research to date has investigated this link in autistic samples, nor explored the relationship with RRB.

At present, with the exception of Hwang et al. (2019), studies exploring the relationships between sensory processing differences, IU, anxiety and RRB experienced by autistic people have only included children and have been based on parent report. Furthermore, no study to date has explored the relevance of alexithymia to these relationships. This study aimed to understand these relationships in autistic adults. We hypothesised that there would be a significant direct path between sensory processing differences and ISB and RMB, respectively. We aimed to investigate whether alexithymia, IU and anxiety were individual and serial mediators of the relationship between sensory processing differences and RMB and ISB, respectively. Figure 1 indicates the pathways tested in this study.

**Fig. 1:**
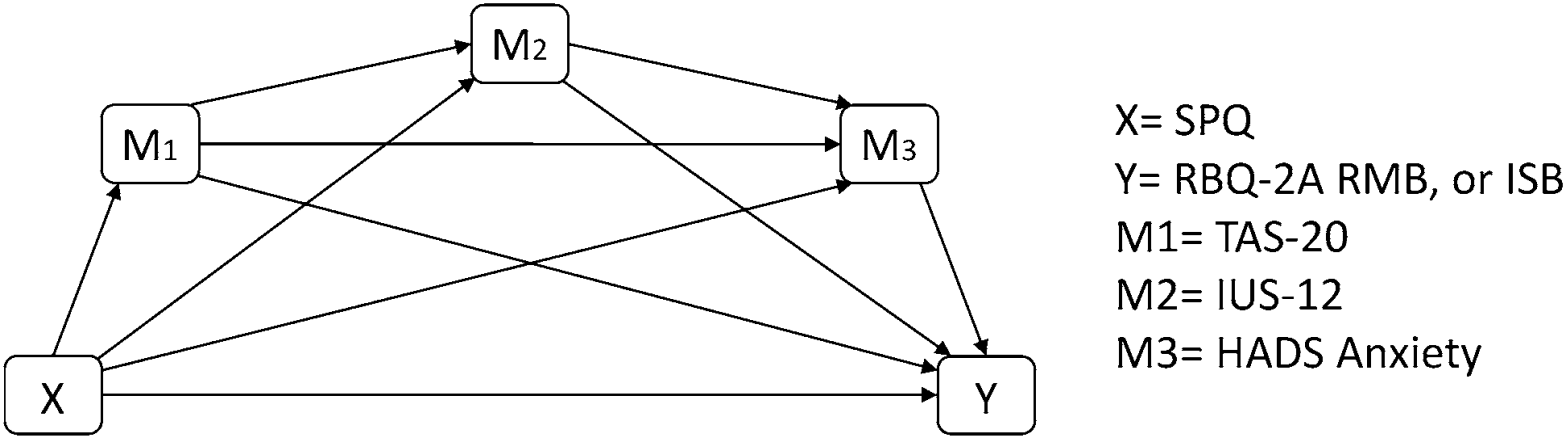
Conceptual diagram of Model 6 (3 serial mediators) in Hayes PROCESS, indicating the direct and indirect pathways tested in this study. X = SPQ; Y = RBQ-2A RMB or ISB; M_1_ = TAS-20; M_2_ = IUS-12; M_3_ = HADS Anxiety

## Methods

### Participants

Data were from 426 participants recruited to the Personalised Anxiety Treatment in Autism (PAT-A^©^) study and recruited via the Adult Autism Spectrum Cohort-UK (ASC-UK; https://research.ncl.ac.uk/adultautismspectrum/), a longitudinal cohort study of autistic adults. Inclusion criteria were autistic people living in the UK aged 18 years and older, who had previously reported a diagnosed or suspected anxiety condition. As in previous research, this sample included some participants who were awaiting autism assessment or suspected they were autistic (Brice et al., 2021; Mason et al., 2018). Table 1 shows sample demographic characteristics. There were no significant differences in key demographic variables, such as age, gender or autistic characteristics between responders and non-responders to this study (see Brice et al., 2021).

**Table 1:** Demographic characteristics of the sample (N = 426)

	N (%)	Mean	SD	Min	Max
Age	426 (100)	42.78	13.89	18	77
*Gender*					
Female	223 (52.35)				
Male	191 (44.84)				
Self-describing/rather not say	12 (2.82)				
*Ethnicity* (N = 412)					
White British	387 (90.85)				
Asian	2 (0.47)				
Black	1 (0.23)				
Mixed/multiple ethnic backgrounds	9 (2.11)				
Other ethnic group	4 (0.94)				
Rather not say	9 (2.11)				
*Highest education*					
Postgraduate degree	75 (17.61)				
Bachelor’s degree	108 (25.35)				
Diploma of higher education	31 (7.28)				
Certificate of higher education	15 (3.52)				
A level	68 (15.96)				
General Certificate of Secondary Education (GCSE)	81 (19.01)				
Basic skills	19 (4.46)				
No formal qualifications	24 (5.63)				
Other	5 (1.17)				
*Employment status* (N = 423)					
Employed without support	156 (36.62)				
Employed with support	9 (2.11)				
Volunteer	36 (8.45)				
Unemployed	183 (42.96)				
Retired	20 (4.69)				
Other	19 (4.46)				
*ASD diagnosis*					
Formal diagnosis	368 (86.38)				
Suspected/awaiting diagnosis	58 (13.62)				
*SRS score*					
Spread of data	379 (88.97)	112.13	25.83	17	185
Meeting cut-off for ASD	364 (96.04)				
Normal	15 (3.96)				
Mild	38 (10.03)				
Moderate	131 (34.56)				
Severe	195 (51.45)				

A favourable ethical opinion was obtained for the PAT-A study from the NHS Health Research Authority (HRA) and Wales REC 5 (ref: 18/WA/0014). All participants provided informed consent.

## Measures

Questionnaires used in this study included:

### *Social Responsiveness Scale-2 (SRS-2; *Constantino & Gruber, 2012)

The SRS-2 is a 65-item self-report questionnaire, measuring areas of social functioning associated with autism, useful for sample characterisation. Items are scored on a four-point Likert scale from 1 to 4, and higher scores indicate more severe symptoms. Scores of 68 or above meet the threshold for autism. Scores ranging from 0 to 67 are normal, 68–84 show mild social reciprocity difficulties, 85–112 show moderate difficulties, and scores of 113 and above indicate severe difficulties.

The SRS-2 has demonstrated excellent internal consistency (α = 0.94–0.96) in autistic and non-autistic populations (Bruni, 2014), and the predictive validity of the adult form is also good, with a specificity level of 0.60 and a sensitivity of 0.86 (Mandell et al., 2011). SRS-2 internal consistency in our sample was good (α = 0.87).

### *Sensory Preferences Questionnaire (SPQ; *Kent, 2014*)*

The SPQ is adapted from the ‘Diagnostic Interview for Social and Communication Disorders’ (DISCO; Wing et al., 2002) to assess sensory processing differences in adult populations. The sensory items from the DISCO were converted into a self-report questionnaire and tested with autistic adults. The SPQ includes 21 items scored using a five-point Likert scale. Total scores range from 21 to 105. A higher score indicates a greater degree of sensory processing difference. There are no reported cut offs for impairment. With ASD populations, Kent (2014) showed good internal consistency (α = 0.89) and external validity, as indicated by large correlations with the Adult/Adolescent Sensory Profile (Brown & Dunn, 2002) sensory seeking (r = 0.69), sensory avoidance (r = 0.75), and sensory avoidance (r = 0.74) quadrants, but not the low registration quadrant (r = − 0.23) of the SPQ. In our sample, internal consistency was good (α = 0.87).

### *Adult Repetitive Behaviour Questionnaire-2 (RBQ-2A; *Barrett et al., 2015*)*

The RBQ-2A is a 20-item questionnaire about RRB, which was adapted from the RBQ-2 (Honey et al., 2007), for use in adult populations. The RBQ-2A scores range from 20 to 60 and there are no reported cut offs for impairment, however, a higher score on the RBQ-2A indicates higher display of RRB. Factor analysis supports a two-factor structure, corresponding to RMB and ISB (Barrett et al., 2015, 2018). The RBQ-2A has good psychometric properties in ASD samples with acceptable-good internal consistency for the two-factor structure (RMB α = 0.70, ISB α = 0.81) in an autistic sample (Barrett et al., 2018). Internal consistency for our sample was acceptable for RMB (α = 0.74) and ISB (α = 0.78).

### *Toronto Alexithymia Scale (TAS-20; *Bagby et al., 1994*)*

The TAS-20 is a self-report questionnaire investigating the presence of alexithymia. The items are scored using a five-point Likert scale from 1 to 5, and total scores range between 20 and 100. A score ≤ 51 indicates alexithymia is not present; a score of 52–60 indicates possible alexithymia, and ≥ 61 indicates alexithymia. The TAS-20 demonstrates good internal consistency (α = 0.81) and good test–retest reliability (r = 0.77) in an undergraduate population (Bagby et al., 1994). TAS-20 internal consistency was good in our sample (α = 0.83).

### *Intolerance of Uncertainty Scale – short form (IUS-12; *Carleton et al., 2007*)*

The IUS-12 is a 12-item scale for which participants rate items regarding uncertainty and avoidance of change on a five-point Likert scale. It is a shortened version of the IUS-27 (IUS-27: Freeston et al., 1994). Total scores range from 12 to 60, and while there are no recommended cut-off scores for impairment, a higher score indicates higher IU. The scale demonstrates excellent internal consistency (α = 0.91) and validity, indicated by large correlations with measures of anxiety (r = 0.54–0.61) in an undergraduate sample (Carleton, 2014; Carleton et al., 2007), and excellent internal reliability (α = 0.93) in a sample referred for anxiety difficulties (McEvoy & Mahoney, 2011). In our sample, internal reliability was excellent (α = 0.91).

### *Hospital Anxiety and Depression Scale (HADS; *Zigmond & Snaith, 1983*)*

The HADS measures experiences of anxiety and depression during the previous week. It is a 14-item scale, 7 corresponding with anxiety and 7 with depression, using a four-point Likert scale between 0 and 3. This study only considered the anxiety items (1, 4, 5, 8, 9, 12, and 13). Total anxiety scores range from 0 to 21; a score of ≤ 7 suggests no anxiety is present, 8–10 indicates mild anxiety, 11–14 indicates moderate anxiety, and 15–21 indicates severe anxiety. In non-autistic clinical samples, the HADS Anxiety has good internal consistency (α = 0.77–0.85) and test–retest reliability (r = 0.79–0.82) (Martin & Thompson, 2002; Roberts et al., 2001). Additionally, in autistic samples, HADS Anxiety shows good internal consistency (α = 0.83), excellent convergent validity, shown through medium, negative correlations with a measure of wellbeing (r = –0.45), and acceptable divergent validity (e.g. medium correlations with HADS Depression, r = 0.34; Uljarević et al., 2018). For our sample, HADS Anxiety internal reliability was good (α = 0.82).

### Procedure

Participants were sent the information sheet and consent forms either via post or electronically, depending on their preference. They then completed the survey either online or using paper copies. Participants provided demographic information and completed an anxiety survey (not reported in this paper), before completing the SRS-2, SPQ, RBQ-2A, TAS-20, IUS-12, and HADS.

### Statistical Analysis

Data were prepared and analysed using IBM SPSS Statistics Version 24 (IBM Corp, 2016). SRS-2 items were reverse scored and then summed to form a total score, according to the manual (Constantino & Gruber, 2012). SPQ items were summed to form a total score. RBQ-2A items on a four-point scale were recoded to a three-point scale, as in previous studies (e.g. Barrett et al., 2015, 2018), to make means and SDs comparable across all items. We used RBQ-2A factor loadings provided by Barrett and colleagues, identified through studies of autistic people (Barrett et al., 2018) to form the RMB (RBQ-2A RMB; ranging from 7 to 21) and ISB (RBQ-2A ISB; ranging from 11 to 33) subscales. TAS-20 items were reverse scored and a total score was calculated from the sum of all scores, according to the manual (Bagby et al., 1994). IUS-12 total score was calculated from the sum of all scores. HADS Anxiety subscale items were recoded and reverse scored according to the manual (Zigmond & Snaith, 1983), before summing the items to produce a HADS Anxiety total score. Online Resource 1 shows full data preparation details. Missing values analysis revealed that between 2.35 and 6.81% of the data were missing from the variables selected for mediation analysis, and Little’s MCAR was nonsignificant (χ^2^ (74) = 62.89, p = 0.818), showing that data are likely to be missing completely at random.

We used the SRS-2 to describe autism characteristics and to compare individuals with a formal and suspected diagnosis before inclusion as one group for analysis. Previous research using this PAT-A sample found no significant difference in autism characteristics between those with an autism spectrum diagnosis and those who suspected they were autistic or were awaiting diagnosis (Brice et al., 2021), which we confirmed in our own subsample of this dataset using the SRS-2 (t (377) = − 0.57, p = 0.5701). Therefore, we used a combined sample.

Before exploring the direct and indirect relationships between SPQ, RBQ-2A, TAS-20, IUS-12, and HADS Anxiety, we conducted Pearson’s correlations to examine the relationships between variables, and between variables and demographic characteristics. Gender was a categorical variable with three non-ordered levels (female, male, self-describing) and the resulting data had unbalanced group sample sizes. Therefore, we conducted Kruskal–Wallis tests between gender and other variables, to determine whether these differed significantly. Mediation analyses were run using Model 6 in the PROCESS v3.3 (Hayes, 2012) macro for SPSS, to test the direct effects (SPQ→RBQ-2A), as well as individual and sequential indirect effects. Figure 1 shows the conceptual model and indicates where in the model each variable was entered. Age and gender (dummy coded) were entered as covariates. The analyses used percentile bootstrapping with 10,000 resamples at 95% upper and lower confidence intervals. Non-significant pathways in the models are indicated by confidence intervals overlapping with zero; effect sizes are indicated by unstandardised B values.

## Results

Descriptive statistics for each variable are shown in Table 2. On the TAS-20, 11.2% of participants showed no signs of alexithymia, 19.9% showed possible alexithymia, and 61.7% scored above the cut-off for alexithymia (7.2% missing). On HADS Anxiety, 9.6% showed no signs of anxiety, 12.9% showed mild signs, 34.1% showed moderate signs, and 40.7% showed severe signs of anxiety (2.8% missing). Other scales did not provide cut off scores, but the mean score as a percentage of the maximum score was 46.6 for the SPQ, 61.1 for RBQ-2A RMB, 73.2 for RBQ-2A ISB, and 74.5 for IUS-12.

**Table 2:** Descriptive statistics for measures of sensory processing, RRB, alexithymia, IU, and anxiety

	N	Min	Max	Mean	SD
SPQ	399	22	95	48.90	13.06
RBQ-2A RMB	408	7	21	12.83	3.15
RBQ-2A ISB	406	12	33	24.17	4.44
TAS-20	397	20	92	64.76	11.99
IUS-12	413	12	60	44.67	9.28
HADS Anxiety	416	1	21	13.48	4.14

Pearson’s correlation showed that SPQ, RBQ-2A RMB, RBQ-2A-ISB, TAS-20, IUS-12 and HADS Anxiety were all positively significantly correlated with one another (Table 3). The strongest correlation with the SPQ was the RBQ-2A RMB, followed closely by ISB. As RBQ-2A RMB and ISB are part of the same measure, large correlations were expected and reported. Although both RBQ-2A RMB and RBQ-2A ISB were positively correlated with IUS-12 and HADS Anxiety, RBQ-2A ISB was more strongly correlated with these measures than RBQ-2A RMB. Age was significantly negatively correlated with RBQ-2A RMB. Indicating that older individuals reported fewer RMB. There were significant differences in score based on gender for SPQ (χ^2^(2) = 12.92, p = 0.002), IUS-12 (χ^2^(2) = 11.84, p = 0.003), and HADS Anxiety (χ^2^(2) = 8.59, p = 0.014). Pairwise comparisons showed that females had significantly higher scores than males on each (SPQ: p = 0.006, IUS-12: p = 0.002, HADS Anxiety: p = 0.030), after Bonferroni adjustment for multiple comparisons, applied by SPSS and using an alpha value of 0.05. No other pairwise comparisons were significant. No gender differences were found for RBQ-2A RMB (χ^2^(2) = 3.04, p = 0.209), RBQ-2A ISB (χ^2^(2) = 1.39, p = 0.498), or TAS-20 (χ^2^(2) = 2.46, p = 0.293).

**Table 3:** Pearson correlations between Age, SPQ, RBQ-2A RMB, RBQ-2 ISB, TAS-20, IUS-12, and HADS Anxiety

	Age	SPQ	RBQ-2A RMB	RBQ-2A ISB	TAS-20	IUS-12
Age	–					
*N*						
SPQ	− 0.02	–				
*N*	*399*					
RBQ-2A RMB	− 0.15^**^	0.65^**^	–			
*N*	*408*	*393*				
RBQ-2A ISB	0.07	0.67^**^	0.61^**^	–		
*N*	*406*	*391*	*398*			
TAS-20	− 0.04	0.37^**^	0.35^**^	0.30^**^	–	
*N*	*397*	*383*	*387*	*388*		
IUS-12	− 0.02	0.39^**^	0.34^**^	0.45^**^	0.41^**^	–
*N*	*413*	*392*	*400*	*399*	*392*	
HADS Anxiety	0.02	0.41^**^	0.37^**^	0.42^**^	0.34^**^	0.50^**^
*N*	*416*	*394*	*404*	*401*	*392*	*407*

*Correlation is significant at 0.05 level

**Correlation is significant at 0.01 level

**Fig. 2:**
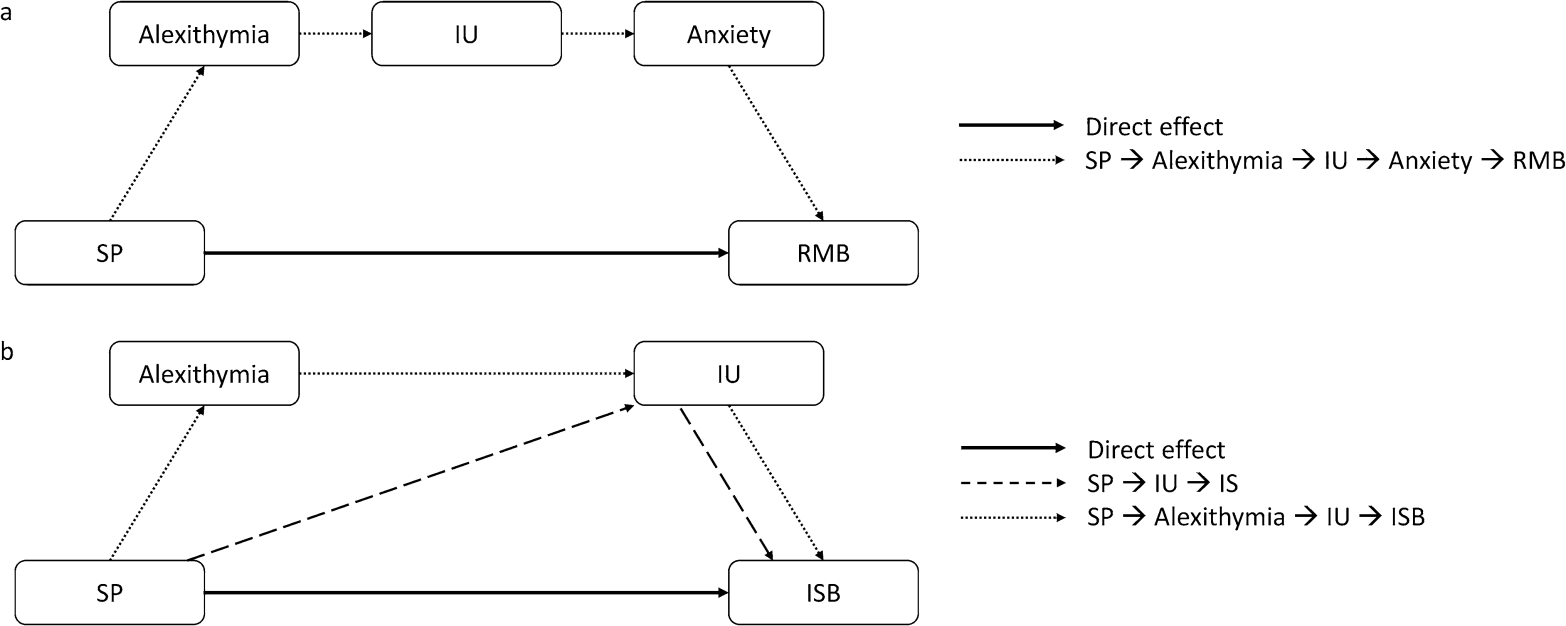
Significant direct and indirect pathways identified through mediation analyses for the relationship between SPQ and RBQ-2A RMB and ISB, respectively.

The mediation output revealed significant direct effects from SPQ to both RBQ-2A RMB and ISB. All significant pathways are displayed in Fig. 2, and all mediation outputs are displayed in Table 4. There was a serial mediating effect through TAS-20 to IUS-12 and HADS Anxiety on the relationship between SPQ and RBQ-2A RMB (Fig. 2a). There were indirect effects of IUS-12 alone, and serial effects of TAS-20 to IUS-12 on the relationship between SPQ and RBQ-2A ISB (Fig. 2b). No other mediating effects were significant.

**Table 4:** Total, Direct and Indirect Effects of TAS-20, IUS-12, and HADS Anxiety as Mediators between SPQ and RBQ-2 ISB and RMB

Predictor	Outcome	Effect		B	SE	LLCI	ULCI
SPQ	RBQ-2A RMB	Total Effect		0.1553*	0.0088	0.1381	0.1725
*N* = *369*		Direct		0.1390*	0.0107	0.1180	0.1600
		Indirect effect(s)	Total	0.0163*	0.0056	0.0056	0.0275
		*Through:*	TAS-20	0.0054	0.0037	− 0.0014	0.0131
			IUS-12	0.0018	0.0033	− 0.0050	0.0083
			HADS Anxiety	0.0048	0.0028	− 0.0001	0.0110
			TAS-20→IUS-12	0.0006	0.0013	− 0.0018	0.0033
			TAS-20→HADS Anxiety	0.0006	0.0006	− 0.0002	0.0021
			IUS-12→HADS Anxiety	0.0022	0.0013	− 0.0001	0.0052
			TAS-20→IUS-12→HADS Anxiety	0.0008*	0.0005	0.0000	0.0021
SPQ	RBQ-2A ISB	Total Effect		0.2254*	0.0125	0.2008	0.2500
*N* = *368*		Direct		0.1881*	0.0151	0.1585	0.2177
		Indirect effect(s)	Total	0.0373*	0.0094	0.0195	0.0563
		*Through:*	TAS-20	− 0.0029	0.0055	− 0.0140	0.0079
			IUS-12	0.0215*	0.0062	0.0104	0.0345
			HADS Anxiety	0.0061	0.0039	− 0.0008	0.0143
			TAS-20→IUS-12	0.0078*	0.0025	0.0036	0.0133
			TAS-20→HADS Anxiety	0.0007	0.0008	− 0.0003	0.0026
			IUS-12→HADS Anxiety	0.0029	0.0019	− 0.0004	0.0070
			TAS-20→IUS-12→HADS Anxiety	0.0011	0.0007	− 0.0001	0.0027

Indirect effects used bootstrapped SE, LLCI, and ULCI

*LLCI* Lower Level Confidence Interval, *UCLI* Upper Level Confidence Interval, *SE* Standard Error

*Effect is significant at 0.05 level

## Discussion

This is the first study to investigate the mediating effects of alexithymia, IU and anxiety on the relationship between sensory processing differences and RRB in a sample of autistic adults. We found significant positive correlations between sensory processing, RRB, and all potential mediating variables, such that as difficulties in one area increased, so they also increased in other areas. Mediation analyses assessed the direct and indirect pathways between these variables, and consistent with our hypothesis, there was a direct effect between sensory processing and both RMB and ISB. Turning to mediators, the relationship between sensory processing differences and RMB showed one serial mediation, through alexithymia to IU to anxiety. Two mediating pathways were identified for the relationship between sensory processing differences and ISB: IU alone, and alexithymia to IU. Anxiety did not play a role in this relationship. These findings show the importance of considering alexithymia, IU, and anxiety, when offering psychological support to autistic people for sensory processing and RRB.

The significant direct effects observed in this sample of autistic adults between sensory processing differences and both RMB and ISB are consistent with previous research with autistic children (Chen et al., 2009; Glod et al., 2019; Wigham et al., 2015). RRB may, therefore, function as an attempt to maintain or regain sensory homeostasis in response to the discomfort of sub-optimal levels of sensory processing difference (Boyd et al., 2010; Gabriels et al., 2008). Specifically, RMB may manifest to increase stimulation when sensory thresholds are raised and ISB may manifest to control the environment and reduce the level of stimulation when sensory thresholds are lowered.

There were no single mediators of the relationship between sensory processing differences and RMB. We did, however, find serial indirect pathways through alexithymia, to IU, to anxiety. This contrasts with child studies that report that anxiety and RMB are not associated with each other (Lidstone et al., 2014; Rodgers et al., 2012); and that IU and anxiety may not play a significant mediating role in the relationship between sensory processing differences and RMB (Glod et al., 2019). Nevertheless, Wigham et al. (2015) showed total indirect effects (but not serial indirect effects) through IU and anxiety in autistic children, partially supporting the role of anxiety in this relationship. It is possible that RMB offer some short-term amelioration of anxiety for adults, whereas in comparison, child samples have indicated that ISB may be more heavily relied upon to manage higher levels of anxiety (Lidstone et al., 2014; Rodgers et al., 2012). Further investigation is warranted, in order to understand the context that elicits different RRB strategies in response to sensory processing differences, and the level of conscious decision making involved.

Considering the relationship between sensory processing differences and ISB, we identified IU as an individual mediator, and alexithymia to IU as serial mediators. Very little work has explored the relationship between alexithymia and IU directly; nevertheless, our findings are consistent with recent research, which has identified correlations between alexithymia and IU in autistic children and adults (Gaigg et al., 2020; Maisel et al., 2016; Ozsivadjian et al., 2021). Sensory processing differences may lead to differences in emotional response and induce feelings of uncertainty about whether aversive stimuli will be encountered in novel settings, how long sensations will last, or how extreme they will feel. Our results suggest that ISB may be employed in an attempt to narrow sensory input by rendering the surrounding environment as predictable and familiar as possible (Boulter et al., 2014; Lidstone et al., 2014).

Anxiety was not a significant individual or serial mediator in the relationship between sensory processing differences and ISB, despite correlations comparable to other mediators. This finding is contrary to the majority of research with autistic children, which has reported anxiety to be significantly associated with ISB such as routines, rituals, and dislike of change (Factor et al., 2016; Lidstone et al., 2014; Rodgers et al., 2012; Spiker et al., 2011). Moreover, previous child research exploring mediators of sensory processing differences and ISB has shown significant indirect pathways through IU to anxiety (Glod et al., 2019; Wigham et al., 2015). It must be noted that much of this research was conducted with samples different to our own (smaller samples; autistic children; different gender profile) and, as with other constructs, it is possible that the role and relationships vary as a result.

We can postulate several conclusions from these findings. Perhaps most important is the novel finding that alexithymia plays an important mediating role in the relationship between sensory processing differences and RRB. It appears that the mediating role of alexithymia is linked to IU, but not directly to anxiety. Indeed, when alexithymia and anxiety are associated with each other, this is also in conjunction with IU. This contrasts with previous research with autistic adults by Maisel et al. (2016), who found that, while alexithymia significantly predicted IU, IU did not explain the relationship between autism symptoms and anxiety once alexithymia and emotional acceptance were included in the model. This study did not investigate serial indirect effects though, potentially masking the role of IU in the relationship between alexithymia and anxiety. Alternatively, inclusion of emotional acceptance may mitigate the role of IU in experiences of anxiety. Another factor to consider is emotion regulation (Morie et al., 2019). However, these constructs depend on the experience of emotions being present initially, and there is a degree of semantic overlap in these constructs that could be better understood if exploring the contribution of these variables to the relationship between sensory processing differences and RRB in future.

We can also conclude that IU is important in mediating the relationship between sensory processing differences and RRB. IU is a multidimensional construct, indicating two broad constructs; the first construct reflects adverse reactions to uncertainty, and an active pursuit of predictability relating to the future, and the second construct represents an inability to act in the face of uncertainty (Berenbaum et al., 2008; Birrell et al., 2011). This uncertainty paralysis has been likened to the freeze response to threat in the fight-flight-freeze system (Birrell et al., 2011). A small amount of research has identified significant associations between measures of anxiety and tonic immobility (or the freeze response) suggesting a pathway by which this facet of IU and anxiety meet (Sagliano et al., 2014; Schmidt et al., 2008). In relation to this, an important finding from our results is that RMB may occur as a coping mechanism specifically in response to sensory processing-related anxiety (although this was found in conjunction with alexithymia and IU). Considering the role of anxiety in relation to the uncertainty paralysis construct that constitutes part of IU, it may be that two pathways exist. The desire for predictability may result in the employment of ISB, whereas paralysis caused by uncertainty may result in anxiety (‘freezing’ in response to threat) and lead to RMB as an avoidant strategy to block out other input. We did not explore IU dimensions in our study, as the IUS-12 is better understood as a unidimensional measure (e.g. Bottesi et al., 2019; Hale et al., 2016; Shihata et al., 2018), so we were not able to test this hypothesis. In future, it would be interesting to use a different measure of IU to determine how the underlying constructs influence mediating relationships between sensory processing and RRB.

One of the major strengths of our study is our large and heterogeneous sample. Despite the greater prevalence of autism diagnoses in males (Loomes et al., 2017), our sample was well balanced, constituting 45% males and 52% females. This can be considered both a strength, as we were able to explore these findings in relation to both genders, and a potential limitation, given that our sample may not be representative of the autistic population. Nevertheless, our sample fits with survey research more broadly, where there is a response rate bias towards females (Sax et al., 2008), and also with online survey research among autistic university students specifically, where similar ratios have been observed (Ames et al., 2016; Anderson et al., 2018; Gelbar et al., 2014). Another strength of our sample is the wide range SRS-2 scores, which allow us to measure the relationship between our variables across the autism spectrum, strengthening our ability to generalise to a wider range of autistic individuals. However, our results cannot be generalised beyond our sample demographic (e.g. to individuals with intellectual difficulties), and it must be acknowledged that our sample may be predisposed towards those with an interest in mental health (Rubenstein & Furnier, 2021). However, responders and non-responders to PAT-A from ASC-UK did not differ on key demographic variables, including autistic characteristics (Brice et al., 2021), supporting the internal validity of our results.

We must also consider limitations of the measures used in this study. The SPQ (Kent, 2014) offers psychometric evidence for a single factor, therefore we were not able to replicate previous findings by exploring sensory hypo- and hyper-reactivity. While the stability of the factor structures is debatable, previous research with children has used parent-report measures such as the Short Sensory Profile (McIntosh et al., 1999) to investigate specific relationships between sub-factors of sensory processing differences and both ISB and RMB, and the role of mediators in these relationships. The understanding of such relationships in adults would aid formulation within psychological therapies, should the clinician and autistic person be able to pinpoint how sensory processing differences present, and the role of RRB that co-occur, while considering the impact of alexithymia, IU and anxiety in those relationships. Anxiety in this sample was measured using the HADS, which, while considered acceptable for use with autistic people (Uljarević et al., 2018), may not be as sensitive as an autism-specific measure due to differential anxiety presentations in autistic people (Brugha et al., 2015; Kerns et al., 2014; Lecavalier et al., 2014; Wigham & McConachie, 2014). A new measure called the Anxiety Scale for Autism-Adults (ASA-A; Rodgers et al., 2020), has been specifically designed for and by autistic people, and it would be interesting to repeat our analyses with this measure. Notably, this measure includes questions that overlap with the IUS-12, which, if not properly managed, could result in criterion confounds.

In conclusion, these findings have important clinical implications, indicating that difficulties with emotional processing, alongside IU and anxiety, should be considered when offering psychological interventions to support autistic people. Additional research into differential relationships with hyper and hypo-sensory sensitivities, the roles of different IU constructs, and the differential purpose of RRB constructs, may advance our understanding of support options further.

## Supplementary Information

Below is the link to the electronic supplementary material.

Supplementary file1 (DOCX 19 KB)

## References

[CR1] Allen R, Davis R, Hill E (2012). The effects of autism and alexithymia on physiological and verbal responsiveness to music. Journal of Autism and Developmental Disorders.

[CR2] Ames ME, McMorris CA, Alli LN, Bebko JM (2016). Overview and evaluation of a mentorship program for university students with ASD. Focus on Autism and Other Developmental Disabilities.

[CR3] American Psychiatric Association. (2013). *Diagnostic and statistical manual of mental disorders (DSM-5®)*: American Psychiatric Pub.

[CR4] Anderson AH, Carter M, Stephenson J (2018). Perspectives of university students with autism spectrum disorder. Journal of Autism and Developmental Disorders.

[CR5] Bagby RM, Parker JDA, Taylor GJ (1994). The twenty-item Toronto Alexithymia scale—I. Item selection and cross-validation of the factor structure. Journal of Psychosomatic Research.

[CR6] Baker AE, Lane A, Angley MT, Young RL (2008). The relationship between sensory processing patterns and behavioural responsiveness in autistic disorder: A pilot study. Journal of Autism and Developmental Disorders.

[CR7] Barrett SL, Uljarević M, Baker EK, Richdale AL, Jones CRG, Leekam SR (2015). The Adult Repetitive Behaviours Questionnaire-2 (RBQ-2A): A self-report measure of restricted and repetitive behaviours. Journal of Autism and Developmental Disorders.

[CR8] Barrett SL, Uljarević M, Jones CRG, Leekam SR (2018). Assessing subtypes of restricted and repetitive behaviour using the Adult Repetitive Behaviour Questionnaire-2 in autistic adults. Mol Autism.

[CR9] Ben-Sasson A, Cermak SA, Orsmond GI, Tager-Flusberg H, Kadlec MB, Carter AS (2008). Sensory clusters of toddlers with autism spectrum disorders: Differences in affective symptoms. Journal of Child Psychology and Psychiatry.

[CR10] Berenbaum H, Bredemeier K, Thompson RJ (2008). Intolerance of uncertainty: Exploring its dimensionality and associations with need for cognitive closure, psychopathology, and personality. Journal of Anxiety Disorders.

[CR11] Bird G, Cook R (2013). Mixed emotions: The contribution of alexithymia to the emotional symptoms of autism. Translational Psychiatry.

[CR12] Birrell J, Meares K, Wilkinson A, Freeston M (2011). Toward a definition of intolerance of uncertainty: A review of factor analytical studies of the Intolerance of Uncertainty Scale. Clinical Psychology Review.

[CR13] Bottesi G, Noventa S, Freeston MH, Ghisi M (2019). Seeking certainty about Intolerance of Uncertainty: Addressing old and new issues through the Intolerance of Uncertainty Scale-Revised. PLoS ONE.

[CR14] Boulter C, Freeston M, South M, Rodgers J (2014). Intolerance of uncertainty as a framework for understanding anxiety in children and adolescents with autism spectrum disorders. Journal of Autism and Developmental Disorders.

[CR15] Boyd BA, Baranek GT, Sideris J, Poe MD, Watson LR, Patten E (2010). Sensory features and repetitive behaviors in children with autism and developmental delays. Autism Research: Official Journal of the International Society for Autism Research.

[CR16] Brice S, Rodgers J, Ingham B, Mason D, Wilson C, Freeston M (2021). The importance and availability of adjustments to improve access for autistic adults who need mental and physical healthcare: Findings from UK surveys. British Medical Journal Open.

[CR17] Brown C, Dunn W (2002). Adult/adolescent sensory profile: User's manual.

[CR18] Brugha TS, Doos L, Tempier A, Einfeld S, Howlin P (2015). Outcome measures in intervention trials for adults with autism spectrum disorders; a systematic review of assessments of core autism features and associated emotional and behavioural problems. International Journal of Methods in Psychiatric Research.

[CR19] Bruni TP (2014). Test review: Social responsiveness scale–Second edition (SRS-2). Journal of Psychoeducational Assessment.

[CR20] Buhr K, Dugas M (2009). The role of fear of anxiety and intolerance of uncertainty in worry: An experimental manipulation. Behaviour Research and Therapy.

[CR21] Carleton RN (2014). The intolerance of uncertainty construct in the context of anxiety disorders: Theoretical and practical perspectives. Expert Review of Neurotherapeutics.

[CR22] Carleton RN, Norton P, Asmundson G (2007). Fearing the unknown: A short version of the intolerance of uncertainty scale. Journal of Anxiety Disorders.

[CR23] Chen YH, Rodgers J, McConachie H (2009). Restricted and repetitive behaviours, sensory processing and cognitive style in children with autism spectrum disorders. Journal of Autism and Developmental Disorders.

[CR24] Constantino, J. N., & Gruber, C. P. (2012). *Social responsiveness scale: SRS-2*: Western Psychological Services Torrance, CA.

[CR25] Crane L, Goddard L, Pring L (2009). Sensory processing in adults with autism spectrum disorders. Autism.

[CR26] Cuccaro ML, Shao Y, Grubber J, Slifer M, Wolpert CM, Donnelly SL (2003). Factor analysis of restricted and repetitive behaviors in autism using the autism diagnostic interview-R. Child Psychiatry and Human Development.

[CR28] Factor RS, Condy EE, Farley JP, Scarpa A (2016). Brief report: Insistence on sameness, anxiety, and social motivation in children with autism spectrum disorder. Journal of Autism and Developmental Disorders.

[CR29] Freeston MH, Rhéaume J, Letarte H, Dugas MJ, Ladouceur R (1994). Why do people worry?. Personality and Individual Differences.

[CR30] Gabriels R, Agnew J, Miller L, Gralla J, Pan Z, Goldson E (2008). Is there a relationship between restricted, repetitive, stereotyped behaviors and interests and abnormal sensory response in children with autism spectrum disorders?. Research in Autism Spectrum Disorders.

[CR31] Gaigg SB, Flaxman PE, McLaven G, Shah R, Bowler DM, Meyer B (2020). Self-guided mindfulness and cognitive behavioural practices reduce anxiety in autistic adults: A pilot 8-month waitlist-controlled trial of widely available online tools. Autism.

[CR32] Gal E, Dyck M, Passmore A (2002). Sensory differences and stereotyped movements in children with autism. Behaviour Change.

[CR33] Gelbar NW, Smith I, Reichow B (2014). Systematic review of articles describing experience and supports of individuals with autism enrolled in college and university programs. Journal of Autism and Developmental Disorders.

[CR34] Glod M, Riby DM, Rodgers J (2019). Short report: Relationships between sensory processing, repetitive behaviors, anxiety, and intolerance of uncertainty in autism spectrum disorder and Williams syndrome. Autism Research: Official Journal of the International Society for Autism Research.

[CR35] Grandin T, Scariano M (1986). Emergence: Labeled autistic.

[CR37] Hale W, Richmond M, Bennett J, Berzins T, Fields A, Weber D (2016). Resolving uncertainty about the intolerance of uncertainty scale-12: Application of modern psychometric strategies. Journal of Personality Assessment.

[CR38] Hayes, A. F. (2012). PROCESS: A versatile computational tool for observed variable mediation, moderation, and conditional process modeling.

[CR39] Hill EL, Berthoz S (2006). Response to "Letter to the Editor: The overlap between alexithymia and Asperger's syndrome", Fitzgerald and Bellgrove. Journal of Autism and Developmental Disorders.

[CR40] Hollocks MJ, Lerh JW, Magiati I, Meiser-Stedman R, Brugha TS (2019). Anxiety and depression in adults with autism spectrum disorder: A systematic review and meta-analysis. Psychological Medicine.

[CR41] Honey E, Leekam S, Turner M, McConachie H (2007). Repetitive behaviour and play in typically developing children and children with autism spectrum disorders. Journal of Autism and Developmental Disorders.

[CR42] Honey E, Rodgers J, McConachie H (2012). Measurement of restricted and repetitive behaviour in children with autism spectrum disorder: Selecting a questionnaire or interview. Research in Autism Spectrum Disorders - RES AUTISM SPECTR DISORD.

[CR43] Hwang YI, Arnold S, Srasuebkul P, Trollor J (2019). Understanding anxiety in adults on the autism spectrum: An investigation of its relationship with intolerance of uncertainty, sensory sensitivities and repetitive behaviours. Autism.

[CR44] IBM Corp. (2016). IBM SPSS Statistics for Windows. (24.0 edn.). Armonk, NY: IBM Corp.

[CR45] Jenkinson R, Milne E, Thompson A (2020). The relationship between intolerance of uncertainty and anxiety in autism: A systematic literature review and meta-analysis. Autism.

[CR46] Joyce C, Honey E, Leekam SR, Barrett SL, Rodgers J (2017). Anxiety, intolerance of uncertainty and restricted and repetitive behaviour: Insights directly from young people with ASD. Journal of Autism and Developmental Disorders.

[CR47] Kapp SK, Steward R, Crane L, Elliott D, Elphick C, Pellicano E (2019). ‘People should be allowed to do what they like’: Autistic adults’ views and experiences of stimming. Autism.

[CR48] Kent, R. G. (2014). *Measuring autism spectrum disorder: Associated features and diagnostic Criteria*. Cardiff University

[CR49] Kern JK, Trivedi MH, Garver CR, Grannemann BD, Andrews AA, Savla JS (2006). The pattern of sensory processing abnormalities in autism. Autism.

[CR50] Kern JK, Trivedi MH, Grannemann BD, Garver CR, Johnson DG, Andrews AA (2007). Sensory correlations in autism. Autism.

[CR51] Kerns CM, Kendall PC, Berry L, Souders MC, Franklin ME, Schultz RT (2014). Traditional and atypical presentations of anxiety in youth with autism spectrum disorder. Journal of Autism and Developmental Disorders.

[CR52] Kientz MA, Dunn W (1997). A comparison of the performance of children with and without autism on the Sensory Profile. American Journal of Occupational Therapy.

[CR53] Kinnaird E, Stewart C, Tchanturia K (2020). Investigating alexithymia in autism: A systematic review and meta-analysis. European Psychiatry.

[CR54] Koerner N, Dugas MJ (2008). An investigation of appraisals in individuals vulnerable to excessive worry: The role of intolerance of uncertainty. Cognitive Therapy and Research.

[CR55] Lecavalier L, Wood JJ, Halladay AK, Jones NE, Aman MG, Cook EH (2014). Measuring anxiety as a treatment endpoint in youth with autism spectrum disorder. Journal of Autism and Developmental Disorders.

[CR57] Lidstone J, Uljarevic M, Sullivan J, Rodgers J, McConachie H, Freeston M (2014). Relations among restricted and repetitive behaviors, anxiety and sensory features in children with autism spectrum disorders. Research in Autism Spectrum Disorders.

[CR58] Liss M, Mailloux J, Erchull MJ (2008). The relationships between sensory processing sensitivity, alexithymia, autism, depression, and anxiety. Personality and Individual Differences.

[CR59] Loomes R, Hull L, Mandy WPL (2017). What is the male-to-female ratio in autism spectrum disorder? A systematic review and meta-analysis. Journal of the American Academy of Child & Adolescent Psychiatry.

[CR60] Maisel M, Stephenson K, South M, Rodgers J, Freeston M, Gaigg S (2016). Modeling the cognitive mechanisms linking autism symptoms and anxiety in adults. Journal of Abnormal Psychology.

[CR61] Mandell DS, Lawer LJ, Branch K, Brodkin ES, Healey K, Witalec R (2011). Prevalence and correlates of autism in a state psychiatric hospital. Autism.

[CR62] Martin CR, Thompson DR (2002). The hospital anxiety and depression scale in patients undergoing peritoneal dialysis: Internal and test–retest reliability. Clinical Effectiveness in Nursing.

[CR63] Mason D, McConachie H, Garland D, Petrou A, Rodgers J, Parr JR (2018). Predictors of quality of life for autistic adults. Autism Research: Official Journal of the International Society for Autism Research.

[CR64] McEvoy PM, Mahoney AEJ (2011). Achieving certainty about the structure of intolerance of uncertainty in a treatment-seeking sample with anxiety and depression. Journal of Anxiety Disorders.

[CR65] McIntosh DN, Miller LJ, Shyu V, Dunn W (1999). Development and validation of the Short Sensory Profile. Sensory profile manual.

[CR66] Milosavljevic B, Carter Leno V, Simonoff E, Baird G, Pickles A, Jones CR (2016). Alexithymia in adolescents with autism spectrum disorder: Its relationship to internalising difficulties, sensory modulation and social cognition. Journal of Autism and Developmental Disorders.

[CR67] Morie KP, Jackson S, Zhai ZW, Potenza MN, Dritschel B (2019). Mood disorders in high-functioning autism: The importance of alexithymia and emotional regulation. Journal of Autism and Developmental Disorders.

[CR68] Neil L, Olsson NC, Pellicano E (2016). The relationship between intolerance of uncertainty, sensory sensitivities, and anxiety in autistic and typically developing children. Journal of Autism and Developmental Disorders.

[CR69] Nemiah, J., Freyberger, H., & PE, S. (1976). Alexithymia: A view of the psychosomatic process. In: O. Hill (Ed.), *Modern trends in psychosomatic research* (Vol. 3, pp. 430–439).

[CR70] O'Brien J, Tsermentseli S, Cummins O, Happe F, Heaton P, Spencer J (2009). Discriminating children with autism from children with learning difficulties with an adaptation of the Short Sensory Profile. Early Child Development and Care.

[CR71] Oakley BFM, Jones EJH, Crawley D, Charman T, Buitelaar J, Tillmann J (2020). Alexithymia in autism: Cross-sectional and longitudinal associations with social-communication difficulties, anxiety and depression symptoms. Psychological Medicine.

[CR72] Ozsivadjian A, Hollocks MJ, Magiati I, Happé F, Baird G, Absoud M (2021). Is cognitive inflexibility a missing link? The role of cognitive inflexibility, alexithymia and intolerance of uncertainty in externalising and internalising behaviours in young people with autism spectrum disorder. Journal of Child Psychology and Psychiatry.

[CR73] Pickard H, Hirsch C, Simonoff E, Happé F (2020). Exploring the cognitive, emotional and sensory correlates of social anxiety in autistic and neurotypical adolescents. Journal of Child Psychology and Psychiatry.

[CR74] Poquérusse J, Pastore L, Dellantonio S, Esposito G (2018). Alexithymia and autism spectrum disorder: A complex relationship. Frontiers in Psychology.

[CR75] Richler J, Bishop SL, Kleinke JR, Lord C (2007). Restricted and repetitive behaviors in young children with autism spectrum disorders. Journal of Autism and Developmental Disorders.

[CR76] Risi S, Lord C, Gotham K, Corsello C, Chrysler C, Szatmari P (2006). Combining information from multiple sources in the diagnosis of autism spectrum disorders. Journal of the American Academy of Child & Adolescent Psychiatry.

[CR77] Roberts SB, Bonnici DM, Mackinnon AJ, Worcester MC (2001). Psychometric evaluation of the Hospital Anxiety and Depression Scale (HADS) among female cardiac patients. British Journal of Health and Psychology.

[CR78] Rodgers J, Farquhar K, Mason D, Brice S, Wigham S, Ingham B (2020). Development and initial evaluation of the anxiety scale for autism-adults. Autism in Adulthood.

[CR79] Rodgers J, Glod M, Connolly B, McConachie H (2012). The relationship between anxiety and repetitive behaviours in autism spectrum disorder. Journal of Autism and Developmental Disorders.

[CR80] Rubenstein E, Furnier S (2021). #Bias: The opportunities and challenges of surveys that recruit and collect data of autistic adults online. Autism Adulthood.

[CR81] Sagliano L, Cappuccio A, Trojano L, Conson M (2014). Approaching threats elicit a freeze-like response in humans. Neuroscience Letters.

[CR82] Salminen JK, Saarijärvi S, Aärelä E, Toikka T, Kauhanen J (1999). Prevalence of alexithymia and its association with sociodemographic variables in the general population of Finland. Journal of Psychosomatic Research.

[CR83] Sax LJ, Gilmartin SK, Lee JJ, Hagedorn LS (2008). Using web surveys to reach community college students: An analysis of response rates and response bias. Community College Journal of Research and Practice.

[CR84] Schmidt NB, Richey JA, Zvolensky MJ, Maner JK (2008). Exploring human freeze responses to a threat stressor. Journal of Behavior Therapy and Experimental Psychiatry.

[CR85] Shihata S, McEvoy PM, Mullan BA (2018). A bifactor model of intolerance of uncertainty in undergraduate and clinical samples: Do we need to reconsider the two-factor model?. Psychological Assessment.

[CR86] South M, Rodgers J (2017). Sensory, emotional and cognitive contributions to anxiety in autism spectrum dsorders. Frontiers in Human Neuroscience.

[CR87] Spiker MA, Lin CE, Van Dyke M, Wood JJ (2011). Restricted interests and anxiety in children with autism. Autism.

[CR88] Turner M (1999). Repetitive behaviour in autism: A review of psychological research. Journal of Child Psychology and Psychiatry.

[CR89] Uljarević M, Lane A, Kelly A, Leekam S (2016). Sensory subtypes and anxiety in older children and adolescents with autism spectrum disorder. Autism Research.

[CR90] Uljarević M, Richdale AL, McConachie H, Hedley D, Cai RY, Merrick H (2018). The Hospital Anxiety and Depression scale: Factor structure and psychometric properties in older adolescents and young adults with autism spectrum disorder. Autism Research.

[CR91] van Steensel FJA, Bögels SM, Perrin S (2011). Anxiety disorders in children and adolescents with autistic spectrum disorders: A meta-analysis. Clinical Child and Family Psychology Review.

[CR92] White SW, Oswald D, Ollendick T, Scahill L (2009). Anxiety in children and adolescents with autism spectrum disorders. Clinical Psychology Review.

[CR93] Wigham S, McConachie H (2014). Systematic review of the properties of tools used to measure outcomes in anxiety intervention studies for children with autism spectrum disorders. PLoS ONE.

[CR94] Wigham S, Rodgers J, South M, McConachie H, Freeston M (2015). The interplay between sensory processing abnormalities, intolerance of uncertainty, anxiety and restricted and repetitive behaviours in autism spectrum disorder. Journal of Autism and Developmental Disorders.

[CR95] Williams D (1992). Nobody nowhere: The remarkable autobiography of an autistic girl.

[CR96] Wing L, Leekam SR, Libby SJ, Gould J, Larcombe M (2002). The diagnostic interview for social and communication disorders: Background, inter-rater reliability and clinical use. Journal of Child Psychology and Psychiatry.

[CR97] Zentall SS, Zentall TR (1983). Optimal stimulation: A model of disordered activity and performance in normal and deviant children. Psychological Bulletin.

[CR98] Zigmond AS, Snaith RP (1983). The hospital anxiety and depression scale.

