# Supplementary material for: The Mediating Effects of Alexithymia, Intolerance of Uncertainty, and Anxiety on the Relationship Between Sensory Processing Differences and Restricted and Repetitive Behaviours in Autistic Adults
Source: J Autism Dev Disord. 2021 Oct 13;52(10):4384–96. doi: 10.1007/s10803-021-05312-1 (PMC9508023; doi:10.1007/s10803-021-05312-1)
Supplement: Supplementary file 1 — Supplementary file1 (DOCX 19 KB) [file 10803_2021_5312_MOESM1_ESM.docx]

**The Mediating Effects of Alexithymia, Intolerance of Uncertainty, and Anxiety on the Relationship between Sensory Processing Differences and Restricted and Repetitive Behaviours in Autistic Adults**

Heather L. Moore^1^, Samuel Brice^2,3^, Lauren Powell^1^, Barry Ingham^2,3^, Mark Freeston^1^, Jeremy R Parr^2,3^, Jacqui Rodgers^2^

1. School of Psychology, 4.28, Dame Margaret Barbour Building, Wallace Street, Newcastle upon Tyne, NE2 4DR.
2. Population Health Sciences Institute, Newcastle University, Sir James Spence Institute, Royal Victoria Infirmary, Level 3, Queen Victoria Road, Newcastle upon Tyne, NE1 4LP, UK.
3. Cumbria, Northumberland, Tyne & Wear NHS Foundation Trust, St Nicholas Hospital, Jubilee Road, Newcastle upon Tyne, NE3 1XT

**Corresponding Author Contact Information**

Heather L. Moore

[heather.moore@newcastle.ac.uk](mailto:heather.moore@newcastle.ac.uk)

**Online Resource 1: Data Preparation Details**

Data were prepared and analysed using IBM SPSS Statistics Version 24 (IBM Corp 2016). SPQ items were summed to form a total score. On the RBQ-2A, items 1-5 and 13-19 were originally scored on a four-point scale; however, as in previous studies e.g. (Barrett et al. 2015; Barrett et al. 2018), the four-point scale was collapsed and recoded to a three-point Likert scale (1=1, 2=2, 3=3, 4=3) to make means and SDs comparable across all items. We used the RBQ-2A factor loadings provided by Barrett and colleagues, identified through studies of autistic people (Barrett et al. 2018), to form the RBQ factors. Questions 1-6 and 10 comprised the Repetitive Motor Behaviour factor (RBQ-2A RMB), and questions 7-9, 11-17, and 19 comprised the Insistence on Sameness factor (RBQ-2A ISB). Items corresponding to each factor were summed to produce total scores; the RBQ-2A RMB ranged from 7-21; the RBQ-2A ISB score ranged from 11-33. Neither items 18 or 20 loaded significantly on to either factor (Barrett et al. 2018), and were excluded from summed scores. TAS-20 items 4, 5, 10, 18 and 19 were reverse scored and a total score was calculated from the sum of all scores, according to the manual (Bagby et al. 1994). IUS-12 total score was calculated from the sum of all scores. The HADS Anxiety subscale (items 1, 4, 5, 8, 9, 12 and 13) was used in the mediation model. According to the manual (Zigmond and Snaith 1983), Items 4 and 13 were recoded (1=0, 2=1, 3=2, 4=3) and items 1, 5, 8, 9 and 12 were reverse coded, before summing the items to produce a HADS Anxiety total score.

**References**

Bagby, R. M., Parker, J. D. A., & Taylor, G. J. (1994). The twenty-item Toronto Alexithymia scale—I. Item selection and cross-validation of the factor structure. *Journal of Psychosomatic Research, 38*(1), 23-32, doi:https://doi.org/10.1016/0022-3999(94)90005-1.

Barrett, S. L., Uljarević, M., Baker, E. K., Richdale, A. L., Jones, C. R. G., & Leekam, S. R. (2015). The Adult Repetitive Behaviours Questionnaire-2 (RBQ-2A): A Self-Report Measure of Restricted and Repetitive Behaviours. *Journal of autism and developmental disorders, 45*(11), 3680-3692, doi:10.1007/s10803-015-2514-6.

Barrett, S. L., Uljarević, M., Jones, C. R. G., & Leekam, S. R. (2018). Assessing subtypes of restricted and repetitive behaviour using the Adult Repetitive Behaviour Questionnaire-2 in autistic adults. *Mol Autism, 9*(1), 58, doi:10.1186/s13229-018-0242-4.

IBM Corp (2016). IBM SPSS Statistics for Windows. (24.0 ed.). Armonk, NY: IBM Corp.

Zigmond, A. S., & Snaith, R. P. (1983). The Hospital Anxiety and Depression Scale. (Vol. 67, pp. 361-370). United Kingdom: Blackwell Publishing.
